# Bibliometrics and Visual Analysis of Adult-onset Still Disease (1976–2020)

**DOI:** 10.3389/fpubh.2022.884780

**Published:** 2022-06-15

**Authors:** Bowen Xu, Jian Wang, Xiaoying Meng, Binghao Bao

**Affiliations:** ^1^Department of Oncology, Guang'anmen Hospital, China Academy of Chinese Medical Sciences, Beijing, China; ^2^Graduate School, Beijing University of Chinese Medicine, Beijing, China; ^3^Department of Nephrology, Dongzhimen Hospital, Beijing University of Chinese Medicine, Beijing, China; ^4^Graduate School, China Academy of Chinese Medical Sciences, Beijing, China

**Keywords:** Adult-onset Still Disease (AoSD), bibliometrics, research trend, VOSviewer, Citespace

## Abstract

**Background:**

Adult-onset Still Disease (AoSD) is a rare disorder without standardized diagnostic criteria. People are paying more and more attention to its research. At present, no published studies have assessed the AoSD field using bibliometric tools. This study aimed to analyze research hotspots and frontiers through bibliometrics to provide a scientific and accurate reference for new and existing researchers.

**Methods:**

Data were obtained from the Web of Science core database and analyzed by CiteSpace, VOSviewer, and Microsoft Excel.

**Results:**

Involving 86 countries and regions, a total of 11,121 authors published 2,199 articles in 676 journals. These studies were published from 1976 to 2020. The United States published the most related articles (397). The United States, France, Italy, and Germany were the top four countries with a high H-index. Authors and institutions with high number of published articles and high citations are mainly located in France and Italy. High-frequency keywords include classification, criteria, diagnosis, and therapy method. Keyword clustering covers the connection between AoSD and rheumatoid arthritis, disease diagnosis, classification, and risk factors.

**Conclusions:**

The research on AoSD focuses on the diagnosis and differential diagnosis of the disease. Targeted therapy will become a research hotspot in the future, and relevant clinical research needs to appropriately expand the sample size and improve the credibility of the conclusions. The data reported in this study can serve as a useful resource for researchers studying AoSD.

## Introduction

Adult-onset Still Disease (AoSD) is a systemic autoinflammatory disease. In 1971, Bywaters analyzed the clinical and laboratory characteristics of 14 adult patients. He found that these characteristics were very similar to systemic-onset juvenile idiopathic arthritis (SoJIA), a condition also known as Still disease. From these observations, he named the disease AoSD (1).

The incidence of AoSD is low. Furthermore, at present, there is no unified understanding of the incidence and prevalence of the disease in different populations in the world. A French study showed that the incidence of AoSD is about 0.16 per 1,00,000 people (2). In Japan, the incidence of AoSD in men and women is 0.22–0.34 per 1,00,000 patients, respectively (3). Additionally, AoSD can occur in any age group and race; however, it is more prevalent in young (15–25 years) and middle-aged (36–46 years) people.

The main characteristics of AoSD include recurrent fever, joint pain or arthritis, skin lesions, hepatosplenomegaly, enlarged lymph nodes, abnormal liver function, increased peripheral blood leukocytes, a high neutrophil ratio, increased C-reactive protein, an enhanced erythrocyte sedimentation rate, and a high concentration of serum ferritin.

Multiple factors such as heredity, infection, and immune dysfunction may be related to the pathogenesis of AoSD; however, the exact mechanism or causes remain unknown. The human leukocyte antigen (HLA) genotype is closely related to the pathogenesis of several diseases. Furthermore, HLA-Bw35, HLA-B17, HLA-B18, HLA-B35, HLA-DR4, HLA-DRw6, etc., are thought to be closely related to the onset of several diseases (4–8). Additionally, interleukin-6 (IL-6), interleukin-8 (IL-8), serum amyloid A, and macrophage migration inhibitory factors are hypothesized to affect disease susceptibility (9–12). Given that similar symptoms of infection such as a sore throat appear before the onset of AoSD, we hypothesize that infectious factors are involved in the onset of the disease. In agreement with this hypothesis, the incidence of AoSD is related to a variety of viruses and pathogenic bacteria (13, 14).

Both the innate and adaptive arms of the host immune response are involved in the pathogenesis of AoSD, of which macrophages, neutrophils, and serum cytokines play a key role. Inflammasome activation, like NLRP3 inflammasome (NOD-, LRR-and pyrin domain-containing 3), by pathogen-associated molecular patterns and damage-associated molecular patterns (15, 16) increase interleukin-1β (IL-1β) and interleukin-18 (IL-18) levels by cysteine-containing aspartate proteolytic enzyme-1 (caspase-1) (17–19). This leads to the dysregulation of IL-6, IL-8, interleukin-17, and Tumor Necrosis Factor alpha (TNF-α) levels (20–22) and results in the induction of cytokine storm and, thus, contribute to AoSD pathogenesis.

Bibliometrics is a comprehensive knowledge system that integrates mathematics, statistics, and linguistics. It is widely used to explore development trends and research frontiers in various fields (23). However, there is no bibliometric study for AoSD. Therefore, we conducted a bibliometric study to evaluate all available evidence for AoSD. Here, we aim to provide an up-to-date overview of the status of AoSD with the help of a variety of bibliometric tools.

## Materials and Methods

### Eligibility Criteria

This study was included literatures on AoSD as the research topic. Article types were limited to “article” and “review.” Conference abstracts, letters, expert views, editorial materials, corrections, retractions, and proceedings paper were excluded.

### Search Strategy and Screening

**Figure 1:**
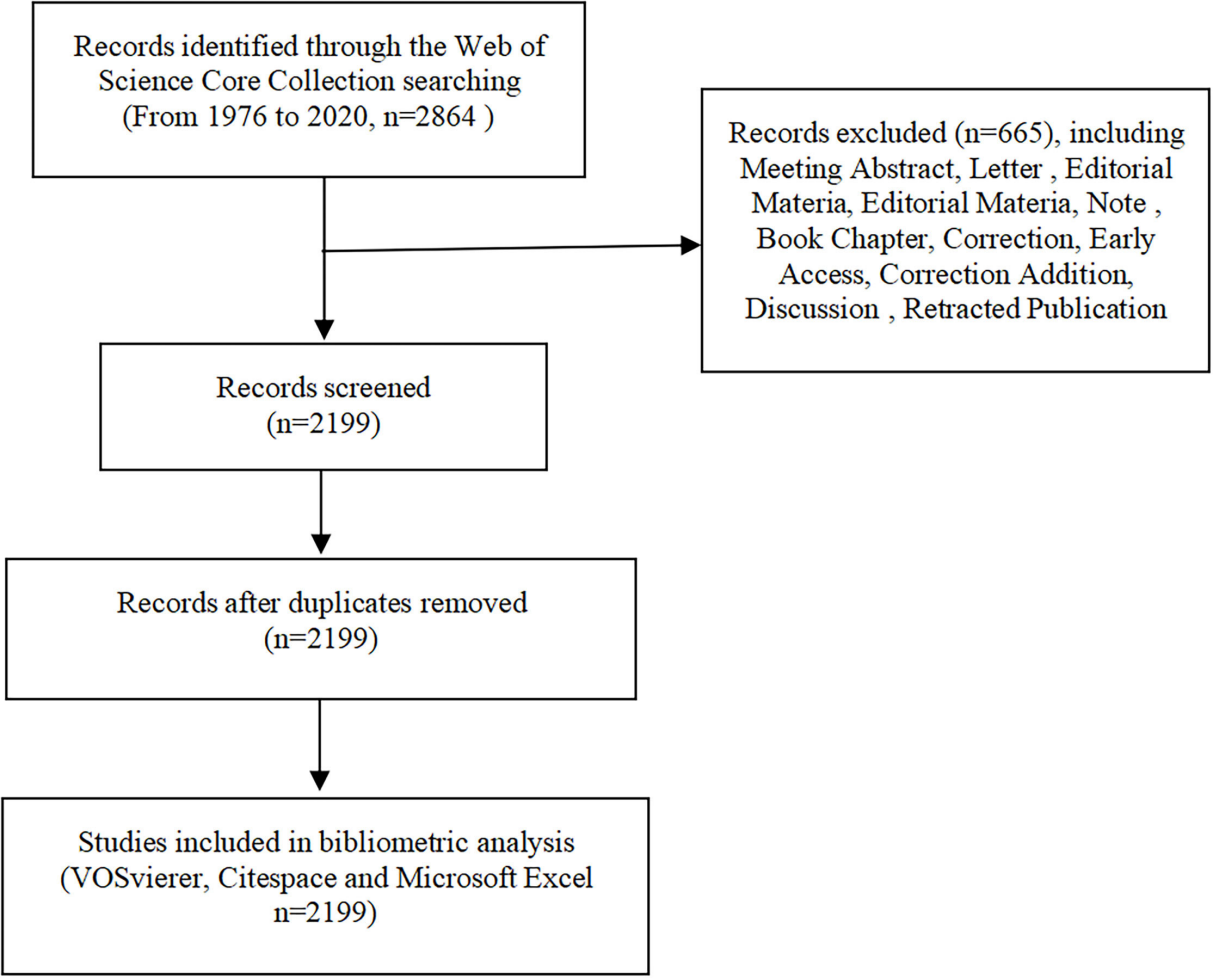
Process of study selection.

All publications included in this study were collected from the Science Citation Index Expanded (SCI-E) through the Web of Science Core Collection (WoSCC) database on October 24, 2021. The search formula was set to TS = (Adult-onset Still Disease), and the period of published studies assessed was from 1976 to 2020, the language restriction was English. Two researchers (BH Bao and BW Xu) cross-checked the included article, and when there were disparities, the third researcher (XY Meng) made a final decision. The process of study screening is shown in Figure 1.

### Data Analysis Methods

VOSviewer version 1.6.17, Citespace 5.8.R3 and Microsoft Excel 2020 are used for data analysis and visualization of AoSD article studies. VOSviewer and Citespace are bibliometric analysis softwares. These softwares were used for co-word analysis, co-citation analysis, and article coupling analysis. They can visualize the research results and has unique advantages in clustering technology and map display (24). Microsoft Excel was used to draw relevant tables and illustrate the annual trend of publications and references.

## Results

### Temporal Trends of Publications and Citations

**Figure 2:**
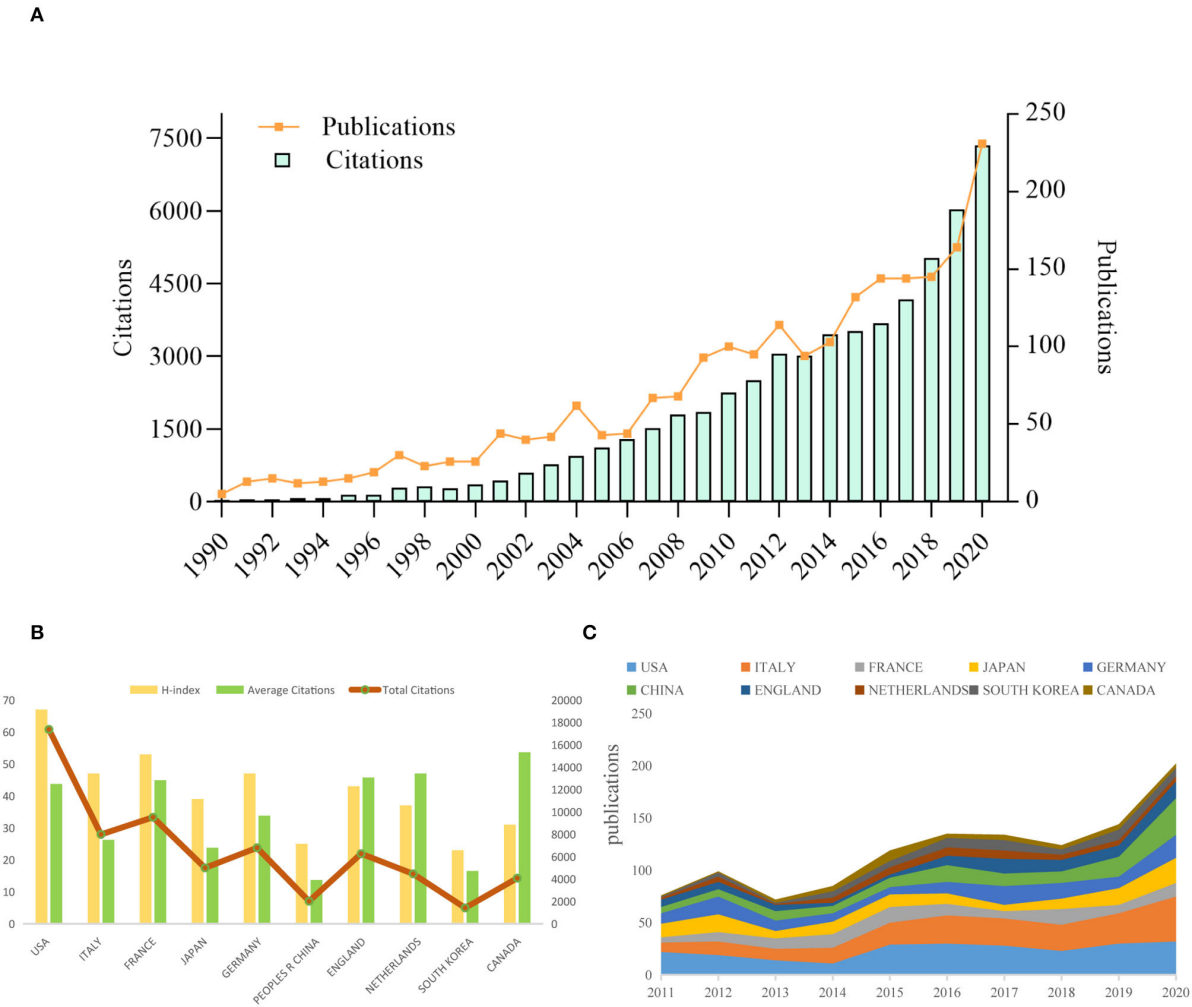
Trends in publications and citations of AoSD research. **(A)** The annual trends of global publications and citations. **(B)** H-index, average citations (citations per article), and total citations of the top 10 countries. **(C)** The temporal trends of publications from the top 10 countries.

By using search strategy, a total of 2,864 published studies and reviews were retrieved. After the removal of duplicate documents, 2,199 articles remained. Changes in the number of publications and citations can reflect the development status of a field and predict future research trends. The total citations and average citations were 63,073 and 28.68, respectively. The H-index was 106. Searching within the WoSCC database, the first available article on AoSD was published in American Family Physician in 1976. In the past 10 years, the number of publications and citations of AoSD article has steadily increased year over year. In 2020, the number of publications and citations of related articles reached peaked at 231 and 11,731. As shown in Figure 2A.

### Country/Region and Institution Characteristics

From 1976 to 2020, scholars from 86 countries and regions have published AoSD research. Among them, scientists from the United States published the most (397, 18.054%). The Top 10 countries with more than 200 publications included Italy (304, 13.824%), France (212, 9.641%), Japan (210, 9.55%), and Germany (201, 9.141%) (Table 1). From the number of publications in the past 10 years, it can be observed that the number of AoSD publications from the Top 10 countries has been steadily increasing (Figure 2C). Furthermore, from the average number of citations within the same period, Canada (53.61), Netherlands (46.98), and England (45.73) are ranked in the Top three and the United States (67), France (47), Italy, Germany (47) were the Top four countries with the highest H-index (Figure 2B and Table 1). Together, these data indicate that the United States, Canada, Brazil, Italy, and other countries have made a substantial contribution to AoSD research.

**Table 1:** Top 10 countries by publications, citations, and centrality.

**Rank**	**Country**	**Publications**	**% of 2,199**	**Total citations**	**Average citations**	**H-index**
1	United States	397	18.054	17,372	43.76	67
2	Italy	304	13.824	7,986	26.27	47
3	France	212	9.641	9,519	44.9	53
4	Japan	210	9.55	4,995	23.79	39
5	Germany	201	9.141	6,794	33.8	47
6	China	147	6.685	2,016	13.71	25
7	England	137	6.23	6,265	45.73	43
8	Netherlands	95	4.32	4,463	46.98	37
9	South Korea	86	3.911	1,420	16.51	23
10	Canada	76	3.456	4,074	53.61	31

Regarding specific research institutions, we found that 2,588 research institutions have conducted AoSD research. The main institutions by number of publications include Assistance Publique Hopitaux Paris (105 publications), Institut National De La Sante Et De La Recherche Medicale (68 publications) and Sorbonne Universite (53 publications). These data indicate that these three institutions are specialized in the field of AoSD research. Further, it is evident that France occupies a dominant position in the field (Table 2).

**Table 2:** Top 10 institutions distributed by publications and centrality.

**Rank**	**Institution**	**Publications**	**Original country**
1	Assistance Publique Hopitaux Paris	105	France
2	Institut National De La Sante Et De La Recherche Medicale	68	France
3	Sorbonne Universite	53	France
4	Universite de Paris	53	France
5	University of London	48	England
6	Harvard University	43	United States
7	Hopital Universitaire Pitie Salpetriere	40	France
8	Sapienza University Rome	38	Italy
9	University of Padua	35	Italy
10	Charite Medical University of Berlin	33	Germany

### Academic Collaboration

**Figure 3:**
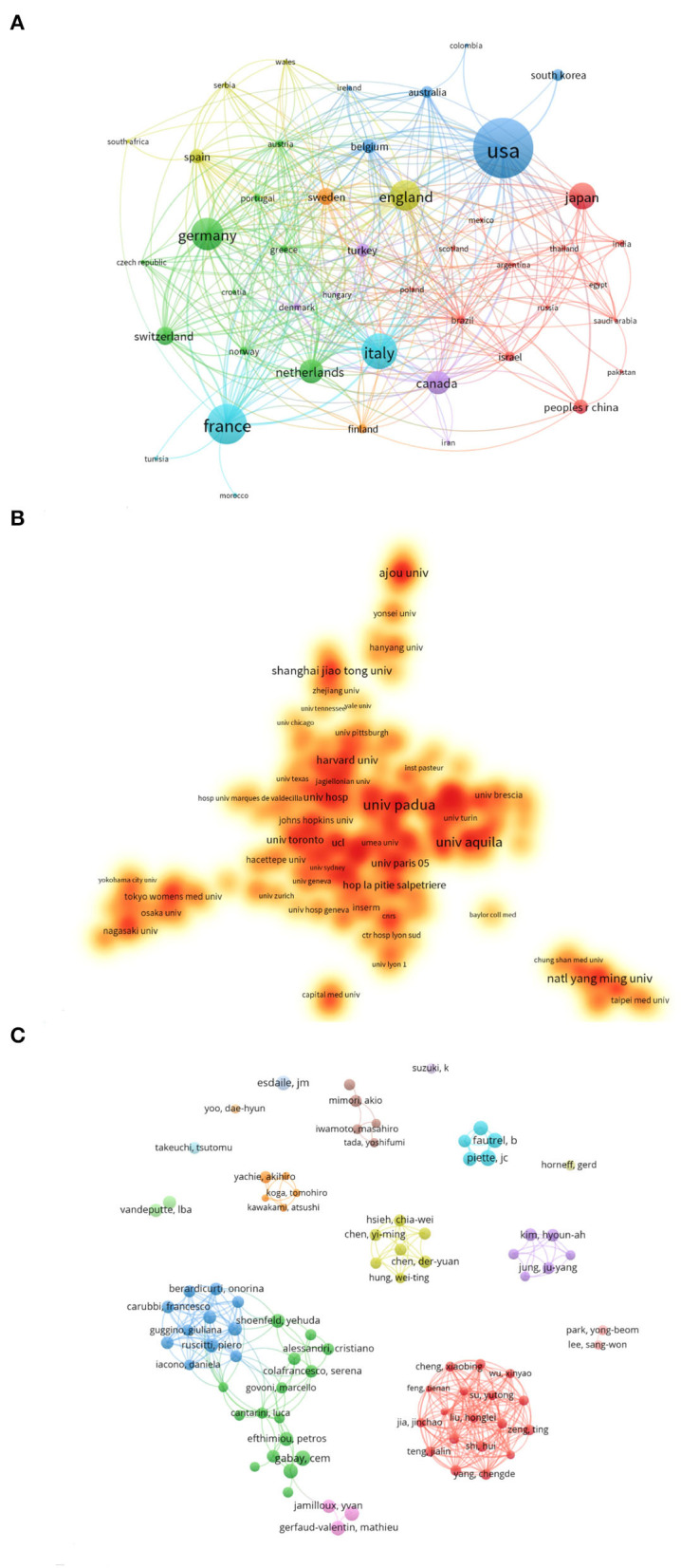
Academic collaboration between different countries/regions, institutions, and authors in AoSD research area. **(A)** Academic collaboration between different countries/regions. **(B)** Collaboration between different institutions. **(C)** Collaboration between different authors.

Academic exchanges and cooperation between different countries/regions, institutions, and authors play an important role in expanding the depth and horizons of research. When parameters of academic cooperation and exchange in AoSD research were assessed, it is evident that collaboration, training, and information exchange occur at multiple levels (Figure 3).

VOSviewer software was used to visualize the collaboration between countries, institutions, and authors (Figure 3A). Each node represents a different country/region. The connection between the nodes represents the cooperative relationship, and the node size represents the number of published studies that have arisen from their collaboration. Of the total published articles, 48 countries/regions published at least five research articles. The United States, Italy, Germany, and France have collaborated the most in AoSD research, which is highly consistent with the observation made for the Top 10 countries in Table 1.

The node connection length reflects the closeness of cooperation between two different institutions. Our analysis revealed that a total of 216 research institutions have published at least five research documents. Circles/nodes in different colors represent a cooperative group. Twelve cooperative groups with the closest cooperation were identified. The University of Padua, the University of L'Aquila, and Harvard University have carried out the most academic cooperation with other research institutions. As shown in Figure 3B.

Co-author analysis revealed a total of 11,121 co-authors in the AoSD field. Giacomelli R (27 articles) has published the most articles, followed by Ruscitti P (26 articles) and Kim Ha (25 articles) (Table 3). Our data indicate that 85 authors have published more than five articles. After clustering these scientists, 16 main cooperative groups were formed (Figure 3C). Among the two largest cooperative groups, Ciccia Francesco and Liu Honglei occupy key positions in the cooperative network, indicating that their research in this field has been significantly cooperative.

**Table 3:** Top 10 authors distributed by publications and citations.

**Rank**	**Author**	**Publications**	**Country**	**Institution**	**Rank**	**Cited author**	**Total citations**	**Average citations**	**H-index**	**Country**	**Institution**
1	Giacomelli R	27	Italy	University of Aquila	1	Fautrel B	1,663	87.53	16	France	Assistance Publique Hopitaux Paris (APHP)
2	Ruscitti P	26	Italy	University of Aquila	2	Piette JC	901	103.7	10	France	Assistance Publique Hopitaux Paris (APHP)
3	Kim Ha	25	South Korea	Ajou University	3	Giacomelli R	753	27.89	16	Italy	University of Aquila
4	Suh CH	24	South Korea	Ajou University	4	Gabay C	740	148	5	Switzerland	University of Geneva
5	Chen DY	21	China	China Medical University	5	Esdaile JM	623	124.8	5	Canada	McGill University
6	Cipriani P	20	Italy	University of Aquila	6	Bourgeois P	616	115.5	6	France	Assistance Publique Hopitaux Paris (APHP)
7	Fautrel B	19	France	Assistance Publique Hopitaux Paris (APHP)	7	Shoenfeld Y	612	61.2	9	Israel	Tel Aviv University Sackler Faculty of Medicine
8	Jung JY	19	South Korea	Ajou University	8	Gerfaud-Valentin M	607	67.44	8	France	University of Claude Bernard Lyon 1
9	Yang CD	17	China	Shanghai Jiao Tong University	9	Seve P	572	67.44	8	France	University of Claude Bernard Lyon 1
10	Giacomelli R	27	Italy	University of L'Aquila	10	Fautrel B	1,663	87.53	16	France	Assistance Publique Hopitaux Paris (APHP)

### Distribution of Co-cited Authors

**Figure 4:**
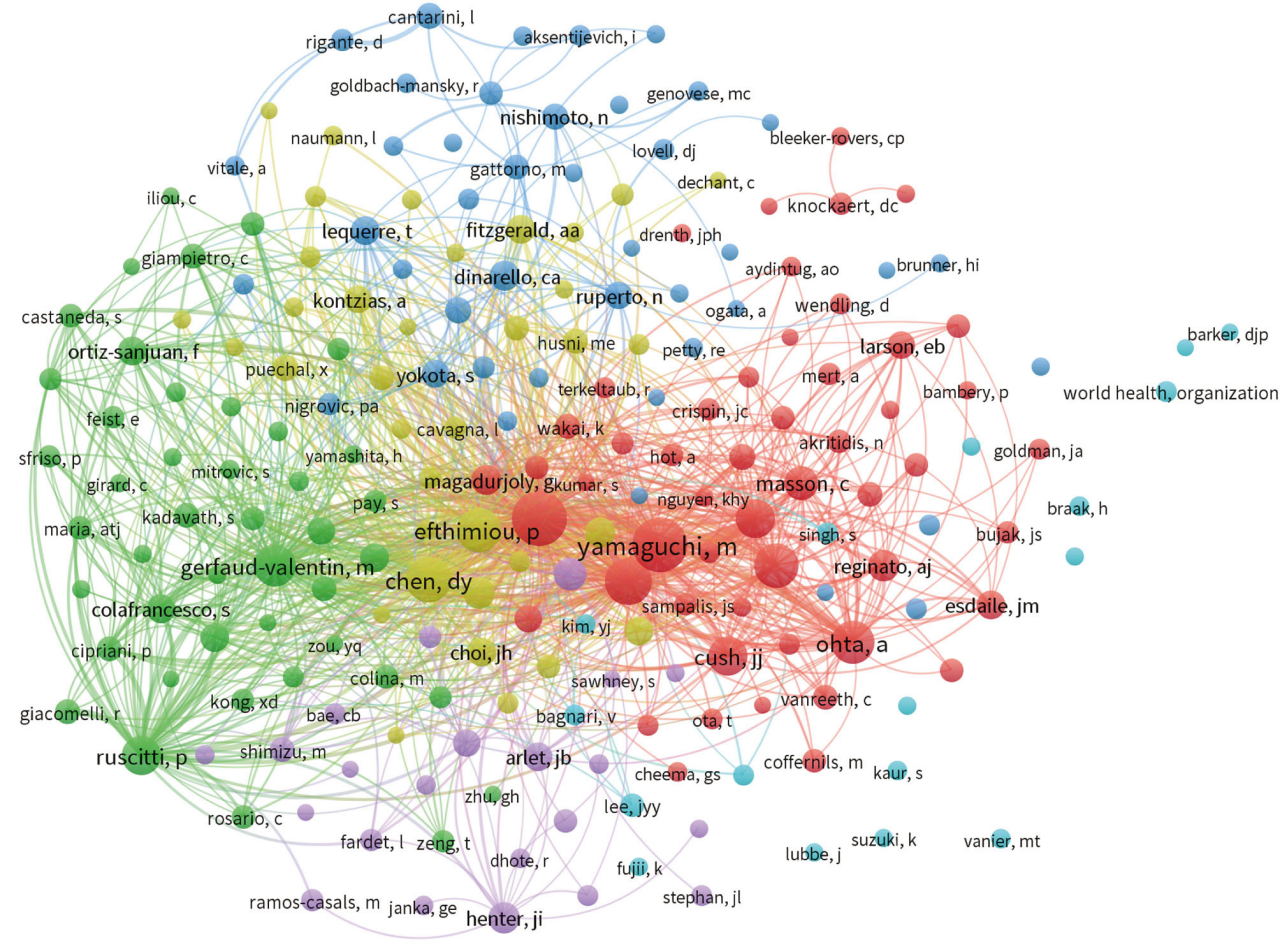
VOSviewer visualization map of co-cited authors devoted to AoSD research.

The Top three most-cited authors were Fautrel B (1,663 citations), followed by Piette JC (901 citations), and Giacomelli R (753 citations). The majority of institutions where the Top 10 authors, by publications and citations, were affiliated were located in France and Italy (Table 3). Yamaguchi M has the highest number of co-citations, followed by Fautrel B, and Pouchot J (Figure 4). These authors have been co-cited more than 380 times, indicating that their AoSD research has received widespread attention.

### Distribution of Journals

**Figure 5:**
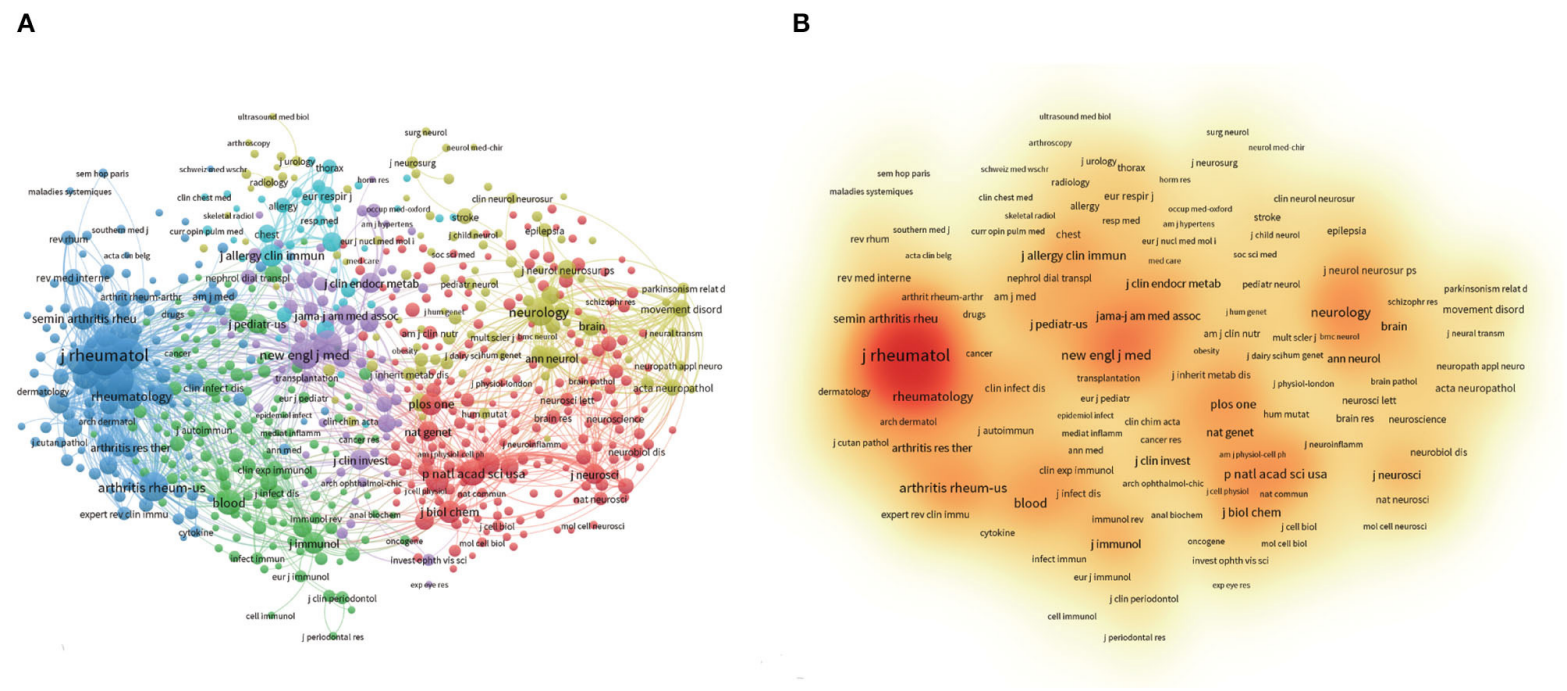
VOSviewer visualization map of most commonly cited journals related to AoSD research. **(A)** Co-citation network of journals. **(B)** The density map of the most commonly cited journals.

The studies and reviews assessed here were published in 676 journals, of which 23 journals published more than 20 articles related to AoSD. The impact factor (IF) and journal quartile were obtained from Journal Citation Reports 2020. The Top four most productive journals are *Clinical Rheumatology* (IF 2.98), *the Journal of Rheumatology* (IF 4.666), *Rheumatology International* (IF 2.631), and *Medicine* (IF 1.889). The Top four journals cited are *the Journal of Rheumatology* (IF 4.666), *Medicine* (IF1.889), *the Annals of the Rheumatic Diseases* (IF 19.103), and *Clinical Rheumatology* (IF 2.98); all of which have been cited more than 1,500 times (Table 4). The Top three most cited journals are *the Journal of Rheumatology* (IF 4.666), *the Annals of the Rheumatic Disease*s (IF 19.103), and *Arthritis & Rheumatology* (IF 10.995) (Figures 5A,B). The majority of the published articles, including the Top 10 most cited journals, are distributed in Q1 or Q2 (Table 4). These data indicate that these journals have a relatively important influence on AoSD research.

**Table 4:** Top 10 journals distributed by publications and citations.

**Rank**	**Journal**	**Publications**	**% of 2,219**	**IF (JCR2020)**	**JIF quartile**	**Journal**	**Total citations**	**Average citations**	**H-index**	**IF (JCR2020)**	**JIF quartile**
1	*Clinical Rheumatology*	79	3.593	2.98	Q3	*Journal of Rheumatology*	3,467	65.45	29	4.666	Q2
2	*Journal of Rheumatology*	53	2.41	4.666	Q2	*Medicine*	1,946	47.54	18	1.889	Q2
3	*Rheumatology International*	46	2.092	2.631	Q3	*Annals of the Rheumatic Diseases*	1,836	63.31	20	19.103	Q1
4	*Medicine*	41	1.864	1.889	Q2	*Arthritis & Rheumatology*	1,731	25.84	17	10.995	Q1
5	*Clinical and Experimental Rheumatology*	39	1.774	4.473	Q2	*Clinical Rheumatology*	1,569	19.87	22	2.98	Q3
6	*Annals of the Rheumatic Diseases*	29	1.319	19.103	Q1	*Journal of Clinical Endocrinology & Metabolism*	1,035	43.08	30	5.958	Q1
7	*Modern Rheumatology*	29	1.319	3.023	Q3	*Rheumatology*	986	46.95	16	7.580	Q1
8	*Revue De Medecine Interne*	24	1.091	0.728	Q4	*Seminars in Arthritis and Rheumatism*	941	42.77	14	5.532	Q2
9	*Internal Medicine*	23	1.046	2.048	Q2	*Journal of Allergy and Clinical Immunology*	895	127.86	7	10.793	Q1
10	*Seminars in Arthritis and Rheumatism*	22	1	5.532	Q2	*Autoimmunity Reviews*	878	73.25	11	9.754	Q1

### Analysis of Highly Cited Articles and Co-cited References

The Top thee co-cited references as follows: “Preliminary criteria for classification of adult stills disease” by Yamaguchi et al. (25) (653 citations). This article attempted to design classification criteria for AoSD. In that paper, AoSD criteria were proposed to be fever, arthralgia, typical rash, and leukocytosis as major, and sore throat, lymphadenopathy and/or splenomegaly, liver dysfunction, and the absence of rheumatoid factor and antinuclear antibody as minor criteria. A positive diagnosis was defined as a patient presenting with five or more criteria including two or more primary criteria (25). The second was “Still's disease in the adult.” by Bywaters (1) (354 citations). In this study, Bywaters first identified an inflammatory disease in young people and observed that the disease was similar to the childhood-onset Still's disease (that is SoJIA). Whilst the exact pathogenesis of the disease remains unclear, there is published homology between AoSD and SoJIA. Third was “Adult stills disease-manifestations, disease course, and outcome in 62 patients” by Pouchot et al. (8) (323 citations). In that study, Pouchot made a preliminary assessment of the clinical and laboratory manifestations, course of disease, outcome, and HLA association of 62 adult patients with Styr's disease from five universities in Canada. He concluded that that the close monitoring of liver function test in patients with AoSD, was important to improve patient outcomes, especially in the early course of the disease and regardless of whether patients use aspirin or other non-steroidal anti-inflammatory drugs (8).

**Figure 6:**
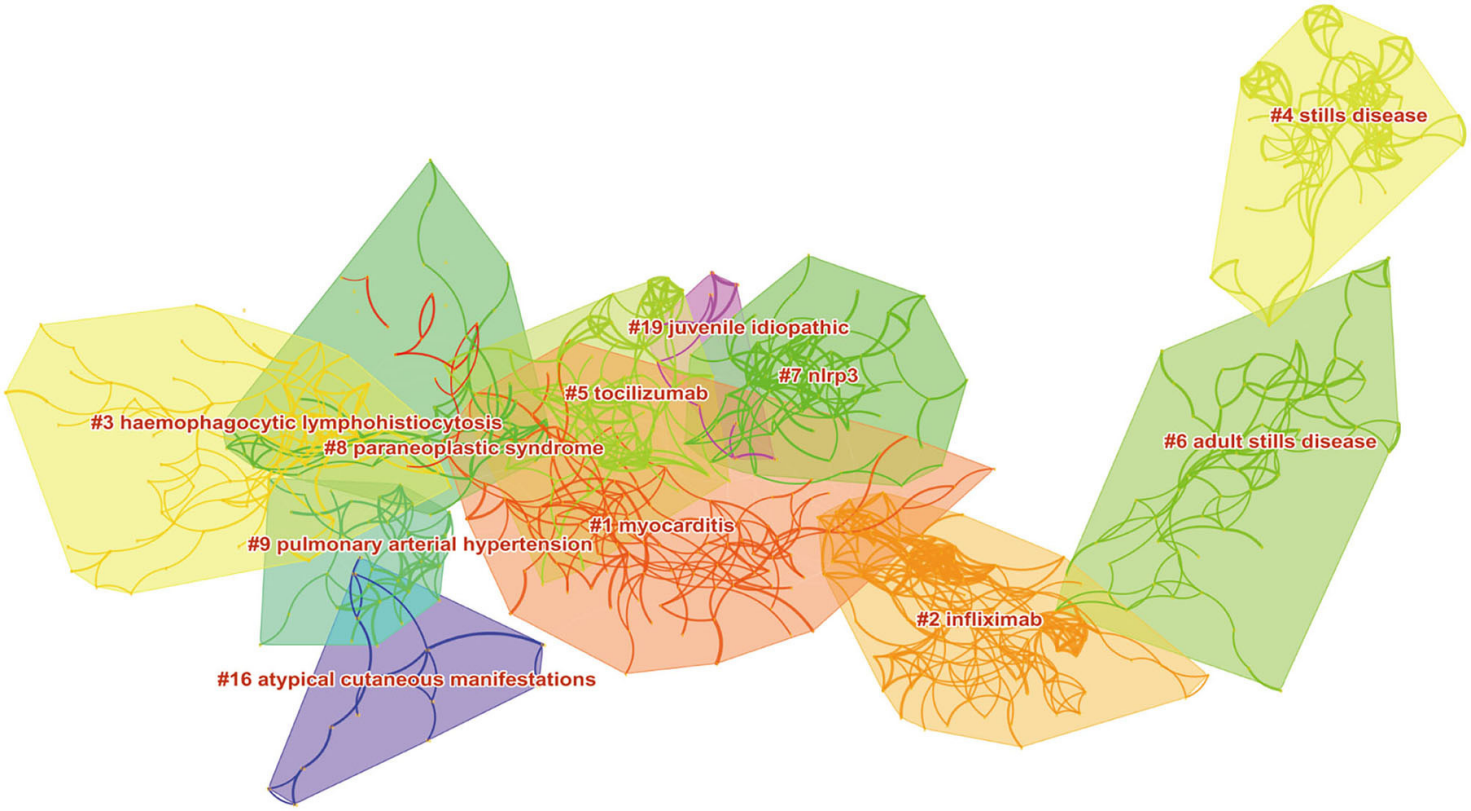
Citespace visualization map of major clusters of co-cited references.

Co-cited references clustering can reflect the frontier direction of research field or unresolved scientific topics to a certain extent, which contains the emerging trend of research. The main clusters of co-cited references on AoSD focus on the treatment (#2, #5), clinical manifestations and accompanying symptoms (#1, #3, #8, #9, #16), differentiation from other rheumatic immune diseases (#4, #6), and autoimmune reactions (#7). As shown in Table 5 and Figure 6.

**Table 5:** Major clusters of co-cited references.

**Cluster ID**	**Cluster label**	**Size**	**S-value**	**Mean (year)**
**#**1	Myocarditis	103	0.885	2016
**#**2	Infliximab	97	0.868	2008
**#**3	Haemophagocytic lymphohistiocytosis	95	0.955	2001
**#**4	Stills disease	80	0.955	2016
**#**5	Tocilizumab	60	0.99	1988
**#**6	Adult stills disease	59	0.933	2010
**#**7	NLRP3	59	0.964	1994
**#**8	Paraneoplastic syndrome	59	0.945	2006
**#**9	Pulmonary arterial hypertension	37	0.94	2013
**#**16	Atypical cutaneous manifestations	31	0.94	2014
**#**19	Juvenile idiopathic	15	1	2014

The Top three articles with the most citations included Yamaguchi et al. (26) (1,081 citations), Pouchot et al. (8) (482 citations) and Gabay et al. (27) (435 citations). The article entitled “IL-1 pathways in inflammation and human diseases” by Gabay et al. (27) has been cited 435 times. This review systematically described published research relating to the IL-1 pathway and inflammation development and treatment. The review found that the human IL-1 receptor antagonist, Anakinra, produced by gene recombination technology affected AoSD (27). As shown in Tables 6, 7.

**Table 6:** Top 10 co-cited reference.

**Rank**	**Author**	**Title**	**Source**	**Citations**
1	Yamaguchi, M	Preliminary criteria for classification of adult stills disease	*Journal of Rheumatology*	653
2	Bywaters EG	Still's disease in the adult	*Annals of the Rheumatic Diseases*	354
3	Pouchot, J	Adult stills disease—manifestations, disease course, and outcome in 62 patients	*Medicine*	323
4	Cush, JJ	Adult-onset stills disease—clinical course and outcome	*Arthritis and Rheumatism*	197
5	Efthimiou P	Diagnosis and management of adult onset Still's disease	*Annals of the Rheumatic Diseases*	175
6	Fautrel, B	Proposal for a new set of classification criteria for adult-onset Still disease	*Medicine*	168
7	Gerfaud-Valentin, M	Adult-onset Still's disease	*Autoimmunity Reviews*	150
8	Chen DY	Proinflammatory cytokine profiles in sera and pathological tissues of patients with active untreated adult onset Still's disease	*Journal of Rheumatology*	132
9	Fautrel, B	Interleukin-1 receptor antagonist (anakinra) treatment in patients with systemic-onset juvenile idiopathic arthritis or adult onset Still disease: preliminary experience in France	*Annals of the Rheumatic Diseases*	128
10	Reginato AJ	Adult onset Stills disease—Experience In 23 patients and literature-review with emphasis on organ failure	*Seminars in Arthritis and Rheumatism*	128

**Table 7:** Top 10 cited articles.

**Rank**	**Author**	**Title**	**Source**	**Citations**
1	Yamaguchi, M	Preliminary criteria for classification of adult stills disease	*Journal of Rheumatology*	1,081
2	Pouchot, J	Adult stills disease—manifestations, disease course, and outcome in 62 patients	*Medicine*	482
3	Gabay, C	IL-1 pathways in inflammation and human diseases	*Nature Reviews Rheumatology*	435
4	Lequerre, T	Interleukin-1 receptor antagonist (anakinra) treatment in patients with systemic-onset juvenile idiopathic arthritis or adult onset Still disease: preliminary experience in France	*Annals of the Rheumatic Diseases*	286
5	Cush, JJ	Adult-onset stills disease—clinical course and outcome	*Arthritis and Rheumatism*	263
6	Gerfaud-Valentin	Adult-onset Still's disease	*Autoimmunity Reviews*	255
7	Fitzgerald, AA	Rapid responses to anakinra in patients with refractory adult-onset Still's disease	*Arthritis and Rheumatism*	251
8	Fautrel, B	Proposal for a new set of classification criteria for adult-onset Still disease	*Annals of the Rheumatic Diseases*	243
9	Efthimiou P	Diagnosis and management of adult onset Still's disease	*Annals of the Rheumatic Diseases*	241
10	Rosario, C	The Hyperferritinemic Syndrome: macrophage activation syndrome, Still's disease, septic shock and catastrophic antiphospholipid syndrome	*BMC Medicine*	235

### Analysis of Keywords

Keywords can also reflect frontiers and hotspots in a particular field. The Top 10 keywords with the highest occurrence frequencies in the article assessed in this study were “classification” (184), “criteria” (177), “rheumatoid-arthritis” (161), “juvenile idiopathic arthritis” (144), “manifestations” (139), “diagnosis” (137), “children” (129), “macrophage activation syndrome” (98), “adults” (94), and “efficiency” (83).

**Figure 7:**
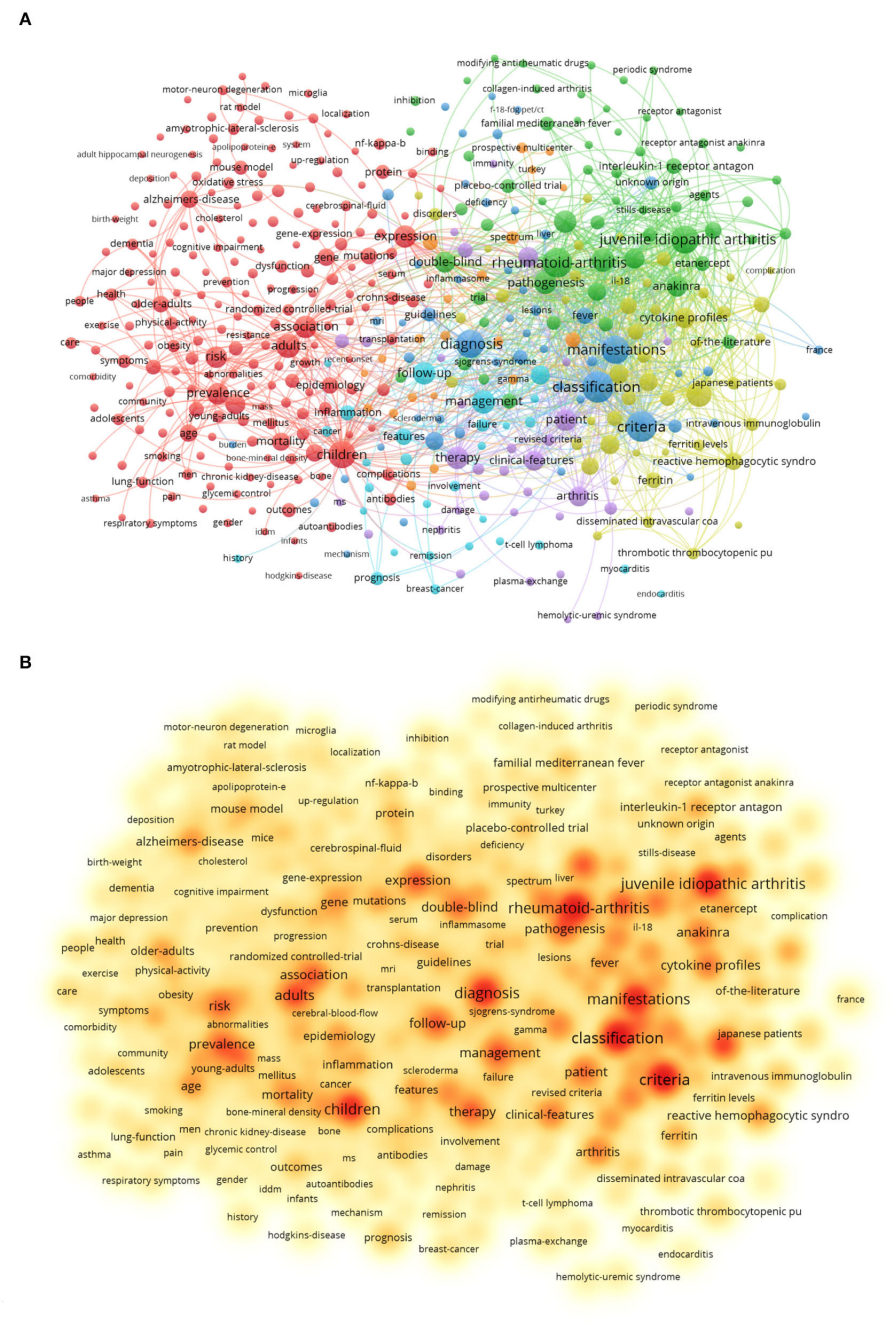
Analysis of all keywords in studies related to AoSD research. **(A)** VOSviewer visualization map of co-occurring keywords. **(B)** The density map of keywords. (The closer the keyword node color is to red, the higher the frequency of its co-occurrence).

**Figure 8:**
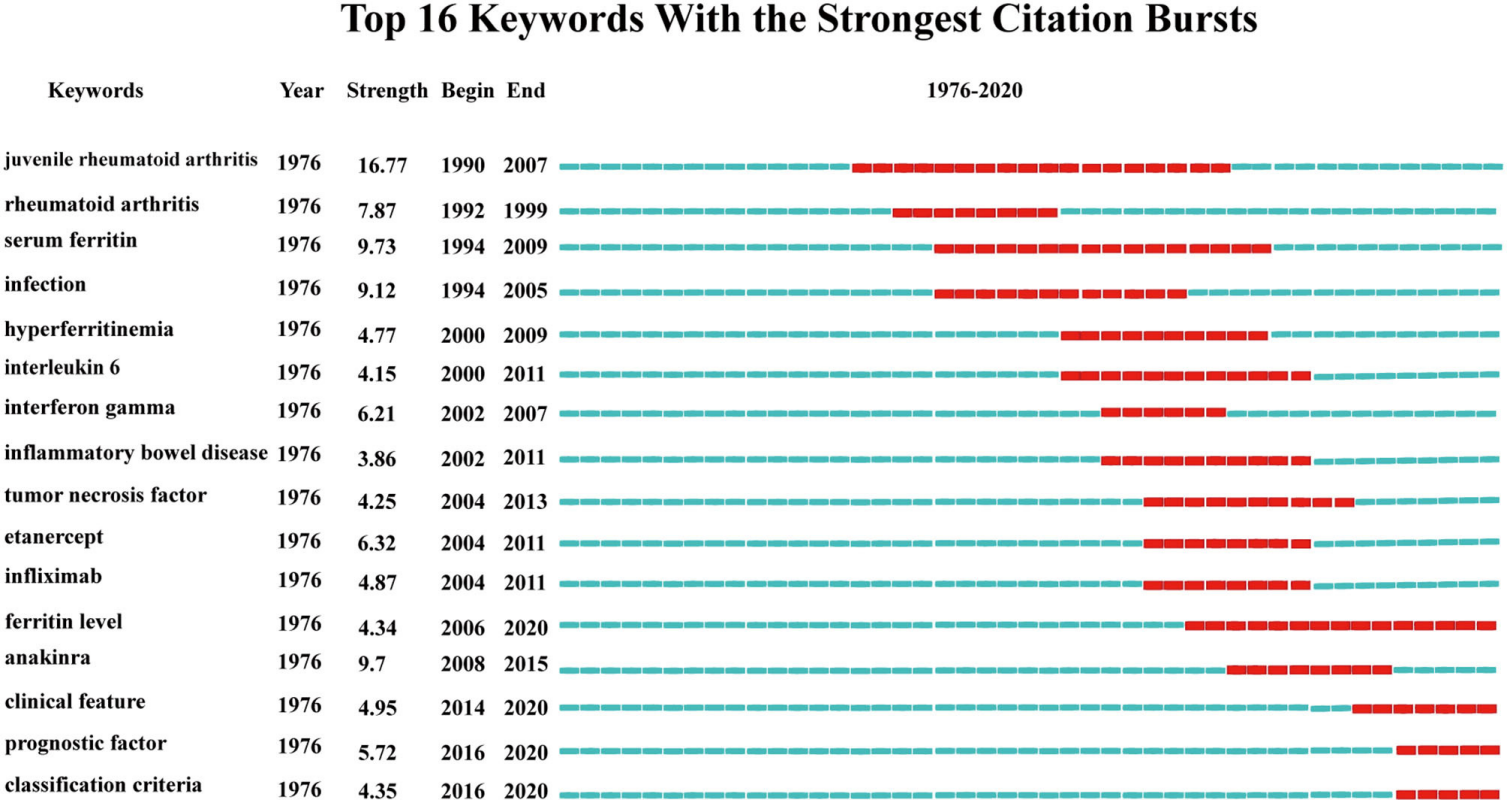
Top16 keywords with the strongest citation bursts.

By clustering keywords with a frequency >5, a total of 446 eligible keywords were clustered into seven groups. Nodes with the same color represent the same cluster (Figure 7A). The clustering theme covers the links with rheumatoid ailment, disease diagnosis, classification, risk factors, and other aspects (Figure 7B). And keywords with a frequency of occurrence higher than 20 were screened for cluster presentation, as shown in Table 8. This is similar to the results of keywords burst. Keyword burst results show that the clinical prognosis judgment of AoSD, disease classification and the biological agents treatment (such as Anakinra) will be the research hotspots in the future (Figure 8).

**Table 8:** Major clusters of keywords.

**Cluster**	**Keyword**	**Number**	**Cluster**	**Keyword**	**Number**
1	Children	129	3	Arthritis	57
1	Disease	124	3	Interleukin-18	49
1	Adults	94	3	Cytokine	39
1	Expression	78	3	Clinical-manifestations	34
1	Prevalence	73	3	pathological tissues	32
1	Risk	70	3	Ferritin	31
1	Risk-factors	66	3	Inflammation	30
1	Mortality	48	3	Marker	30
1	Alzheimer's-disease	43	3	Peripheral-blood	28
1	Age	42	3	Classification criteria	27
1	Gene	41	3	Interferon-gamma	25
1	Epidemiology	38	3	Classification	184
1	Mutations	37	4	Criteria	177
1	Older-adults	36	4	Manifestations	139
1	Proteins	32	4	Experience	54
1	Population	30	4	Features	38
1	Cardiovascular-disease	28	4	Adult-onset	23
1	Natural-history	26	4	Prognosis	21
1	Quality-of-life	25	4	Diagnosis	137
1	Mouse model	24	5	Management	82
1	Women	23	5	Infection	48
1	Physical-activity	21	5	Fever	39
1	Symptoms	21	5	Guidelines	26
2	Rheumatoid-arthritis	161	5	Placebo-controlled trial	23
2	Juvenile idiopathic arthritis	144	5	Therapy	77
2	Efficacy	83	6	Double-blind	67
2	Follow-up	78	6	Systemic-lupus-erythematosus	57
2	Onset stills-disease	76	6	Interleukin-6	24
2	Pathogenesis	75	6	Trial	22
2	Anakinra	62	6	Inflammatory-bowel-disease	21
2	Clinical-features	59	6	Macrophage activation syndrome	98
2	Multicenter	49	7	Tumor-necrosis-factor	61
2	Tocilizumab	35	7	Serum ferritin	55
2	Interleukin-1 receptor antagonist	33	7	Juvenile rheumatoid-arthritis	53
2	Etanercept	30	7	Reactive hemophagocytic syndrome	41
2	Safety	28	7	Hemophagocytic syndrome	40
2	Methotrexate	26	7	Lymphohistiocytosis	26
2	Familial mediterranean fever	24	7	Prognostic-factors	25
2	Infliximab	23	7	Hemophagocytic lymphohistiocytosis	22
2	Spectrum	21	7	Intravenous immunoglobulin	22
3	Cytokine profiles	62			

## Discussion

This study uses a variety of bibliometric software and tools to analyze the published research article on AoSD based on the WoSCC database. Studies have shown that the earliest retrospective article was published in 1976 (28).

By 2020, the annual number of publications and citations of AoSD-related research showed an overall upward trend, thus highlighting the rapid development of AoSD studies and sustained interest in its research. However, as an orphan disease, AoSD has a low incidence in the population with the prevalence rate estimated to be one case per million people in Europe and 10 cases per million people in Japan (2, 3). This low incidence has translated into fewer published studies relative to other rheumatic diseases. From 1976 to 2020, a total of AoSD 2,199 articles were published indicating the growing importance of AoSD research in spite of its low incidence. In addition, compared with other rare immune-related diseases such as Primary Combined Immune Deficiency (26), Chediak-Higashi Syndrome (29), and Chronic Primary Granulomatous Disease (30), which publications were 866, 1,252, and 1,425, respectively. The result indicates that AoSD may attract more interest than these rare diseases in terms of publications. However, compared with common rheumatic immune diseases such as rheumatoid arthritis (more than 190,000 articles) and systemic lupus erythematosus (more than 80,000 articles), the publication count of papers on AoSD is still significantly insufficient.

Through the comprehensive and systematic summary of the research topics, trends, and global research on the impact of AoSD, scholars can quickly and preliminarily understand the current state of AoSD research. The H-index reflects the academic achievements of countries, institutions, or individuals in a certain field. The higher the H-index, the greater the academic influence. From the perspective of regional distribution, the United States is the global leader in terms of both the number of published article and H-index in AoSD-related research. Specifically, AoSD research originating in the United States has significantly contributed to the field. However, in the current study, further analysis of research institutions and individuals revealed that France and Italy may have superior contributing roles to global AoSD research. This is especially valid when assessing the Top 10 authors by the number of publications and citations. Further, these data indicated that no American scholars were found in the Top 10, and French and Italian scholars occupy the majority.

Keyword cluster analysis can highlight current research hotspots. In agreement with the research direction of highly cited articles and co-cited references, our data indicate that the research hotspots of AoSD are mainly related to the identification and diagnosis of the disease. These hotspots may be attributed to the fact that as an orphan disease, the pathogenesis of AoSD is not clear, and multiple factors including heredity, infection, and immune dysfunction may contribute to AoSD development. As an immune-related disease, AoSD is difficult to distinguish from juvenile rheumatoid arthritis and rheumatoid arthritis (RA) in the initial clinical diagnosis.

Additionally, multiple difficulties exist in the diagnosis of AoSD resulting in a lack of a unifying clinical diagnostic gold standard. Traditionally, the diagnostic criteria for AoSD have mainly been based on four main symptoms including fever, arthritis or joint pain, rash, and increased white blood cell and neutrophil counts (14). Fautrel proposed a new diagnostic classification criterion in 2002. His set of eight diagnostic classification criteria included spiking fever ≥39°C, arthralgia, maculopapular rash, transient erythema, pharyngitis, leukocyte count ≥10,000/mm^3^, polymorphonuclear count ≥80%, and glycosylated ferritin ≤20%. Of these criteria, maculopapular rash and leukocyte count ≥10,000/mm^3^ are considered to be secondary, and the remaining six signs are considered to be the primary criteria. Using these parameters, a positive AoSD diagnosis would be defined as a patient presenting with four primary criteria, or three primary criteria +2 secondary criteria. Studies have shown that these diagnostic criteria have a higher sensitivity relative to traditional diagnostic strategies (31). Because the symptoms of AoSD are diverse and systemic, it is necessary to differentiate AoSD from a variety of other diseases such as infections, malignancies, and other chronic rheumatism during diagnosis. These newly proposed criteria may assist with this. There are still a variety of diagnostic and classification criteria (32), but most of the criteria are exclusionary diagnosis. Even after the diagnosis is made, the changes of the disease should be observed at any time during treatment and follow-up. Since the research on AoSD is relatively short and many contents are not yet perfect, developing a diagnostic standard with higher specificity is still a hotspot and difficulty in future research.

Previously, non-steroidal anti-inflammatory drugs, glucocorticoids, and disease-modifying antirheumatic drugs have been used for the treatment of AoSD; however, their curative effect is poor. Further, in ~40% of cases, patient symptoms remain unaffected by traditional treatment (33, 34). Common anti-rheumatic immune drugs such as interferon-γ, etanercept and anakinra are also widely used clinically (35). The activation of innate immunity and the dysregulation of cytokines are important mechanisms contributing to the pathogenesis of AoSD. IL-1β, IL-18, IL-6, and TNF-α are elevated in the serum, skin lesions, and synovial membrane of AoSD patients. The targeted therapy for these factors has been successfully applied in other models of chronic rheumatic disease and may provide a reference for the treatment of AoSD. Many studies have shown that biological agents have good safety and effectiveness in the treatment of refractory AoSD, but the sample size of clinical trials is obviously inadequate (20, 36, 37).

We found that research on targeted therapy for AoSD has been on the rise. As early as 2007, Efthimiou suggested that recent advances in basic immunology have improved our ability to hinder the pathogenic mechanisms associated with AoSD, and discussed the impact of targeted anti-inflammatory cytokine-based targeted therapies on AoSD (14, 38, 39). An increasing number of clinical trials have demonstrated that targeting these proinflammatory cytokines may be effective in controlling AoSD (40). Antagonists of IL-1β and its receptor have been successfully used to treat AoSD (41). Three anti-IL-1β biologics, namely, anakinra, canakinumab, and rilonacept, which are used to control AoSD, are currently available on the market. In the results of this study, Anakinra is one of the current research hotspots, and the publications of papers mentioning AoSD with Anakinra as 227 on WOS data. To our knowledge, a randomized, double-blind, placebo-controlled, multicenter, phase III study to assess the efficacy and safety of anakinra in the treatment of AoSD (https://clinicaltrials.gov/ct2/show/study/NCT03265132), just concluded the update in June 2021, and no relevant literature has been published. In addition, systematic reviews are indicating that the use of IL-1β inhibitors in AoSD has led us to a new era of targeted therapy for AoSD (42). Whilst TNF-α inhibitors, as the first biological agent for the treatment of AoSD, have a good curative effect in chronic inflammatory joint diseases (especially RA), the curative effect in the treatment of AoSD is poor (43). Therefore, we believe that as molecular biology progresses, targeted therapy will play a more beneficial role in AoSD.

Due to the rarity of AoSD, many clinical studies are limited by small sample sizes and are thus underpowered. Furthermore, because of the varying clinical designs among different studies an accurate consensus is difficult to conclude. These are significant obstacles that need to be overcome to improve the credibility of published AoSD studies in the future.

## Limitation

Analysis of AoSD article by Citespace and Vosviewer has some limitations. The first is the deadline for article retrieval by 2020, but as WoSCC continues to update, the number of articles retrieved in this study may be slightly different from the actual number of relevant articles. The Second, due to the possible time lag, the importance of some studies, especially some newly published article, may not be given sufficient attention at present. Finally, the use of single database WOSCC is also a limitation. However, its use can be further substantiated, as it provides more accurate document type labeling than other databases such as Scopus (44). And this research based on bibliometrics still well demonstrates the research hotspot and development trend of AoSD, especially providing reference for young scholars in scientific decision-making.

## Conclusion

This study analyzed AoSD research published from 1976 to 2020. Furthermore, we summarized the regional distribution, journal category, article citation, main cooperative groups, and keyword clustering related to AoSD research. As an understudied, orphan disease, the mechanisms and pathogenesis of AoSD still need to be elucidated. The development of targeted AoSD therapy is a relatively effective and safe. However, existing clinical research samples sizes are small and prone to Type I or Type II errors. It is necessary to further expand the clinical sample size and clarify the safety and effectiveness of biological agents. This study provides key insights into current AoSD research and can be used as a useful resource for researchers in the field to identify hotspots and trends.

## Data Availability Statement

The original contributions presented in the study are included in the article/supplementary material, further inquiries can be directed to the corresponding author/s.

## Author Contributions

BX and BB conceived the study. BX, JW, and XM collected the data. BB re-examined the data. BB and XM analyzed the data. BX wrote the manuscript. JW, BB, and XM reviewed and revised the manuscript. All authors have read and agreed to the published version of the manuscript.

## Conflict of Interest

The authors declare that the research was conducted in the absence of any commercial or financial relationships that could be construed as a potential conflict of interest.

## Publisher's Note

All claims expressed in this article are solely those of the authors and do not necessarily represent those of their affiliated organizations, or those of the publisher, the editors and the reviewers. Any product that may be evaluated in this article, or claim that may be made by its manufacturer, is not guaranteed or endorsed by the publisher.
